# Circulars of Information of the Bureau of Education

**Published:** 1876-01

**Authors:** 


*Circulars of Information of the Bureau of Education,* No. 7, 1875, contains the constitutional provisions in regard to Education in the several states of the American Union.

